# Hæmorrhage Diathesis

**Published:** 1887-09

**Authors:** 


### Hæmorrhagic Diathesis.

*Editor Medical World:*

I have a family under my care who are disposed to bleed to death on any and all occosions offered, no matter how slight the wound: if the skin is merely scratched severe hæmorrhage follows.

About two weeks ago one of the children scratched the lower lip on a pin, and up to this time I have been unable to stop the flow of blood longer than a few hours at a time. Have cauterized the wound, and used all known remedies locally, but to no purpose. As soon as the blood stops flowing the parts begin to swell, and when the parts become full of blood it will start to bleeding and keep it up until the swelling is all out, when the same thing is repeated again; compresses do no good. There are four children in the family, and the same trouble affects each one. Neither of the parents was ever affected in a similar way.

What is the trouble and what is the treatment?

Spencerville, Ohio. J. R. WELCH, M.D.

[Probably strychnine, ergot, and sulphuric acid would assist in stopping the flow. To correct the diathesis, strengthen the tissues by a long administration of the lacto-phosphate of lime. Avoid iron. Reduce the amount of liquids in food to the smallest quantity.—ED.]

